# An fMRI study into emotional processing in Parkinson’s disease: Does increased medial prefrontal activation compensate for striatal dysfunction?

**DOI:** 10.1371/journal.pone.0177085

**Published:** 2017-05-09

**Authors:** Anja J. H. Moonen, Peter H. Weiss, Michael Wiesing, Ralph Weidner, Gereon R. Fink, Jennifer S. A. M. Reijnders, Wim M. Weber, Albert F. G. Leentjens

**Affiliations:** 1 Department of Psychiatry, Maastricht University Medical Center, Maastricht, the Netherlands; 2 Institute for Neuroscience and Medicine (INM-3), Research Centre Jülich, Jülich, Germany; 3 Department of Neurology, University Hospital Cologne, Cologne, Germany; 4 Department of Lifespan Psychology, Open University, Heerlen, the Netherlands; 5 Department of Neurology, Maastricht University Medical Center, Maastricht, the Netherlands; University of Medicine & Dentistry of NJ - New Jersey Medical School, UNITED STATES

## Abstract

**Background:**

Apart from a progressive decline of motor functions, Parkinson’s disease (PD) is also characterized by non-motor symptoms, including disturbed processing of emotions. This study aims at assessing emotional processing and its neurobiological correlates in PD with the focus on how medicated Parkinson patients may achieve normal emotional responsiveness despite basal ganglia dysfunction.

**Methods:**

Nineteen medicated patients with mild to moderate PD (without dementia or depression) and 19 matched healthy controls passively viewed positive, negative, and neutral pictures in an event-related blood oxygen level-dependent functional magnetic resonance imaging study (BOLD-fMRI). Individual subjective ratings of valence and arousal levels for these pictures were obtained right after the scanning.

**Results:**

Parkinson patients showed similar valence and arousal ratings as controls, denoting intact emotional processing at the behavioral level. Yet, Parkinson patients showed decreased bilateral putaminal activation and increased activation in the right dorsomedial prefrontal cortex (PFC), compared to controls, both most pronounced for highly arousing emotional stimuli.

**Conclusions:**

Our findings revealed for the first time a possible compensatory neural mechanism in Parkinson patients during emotional processing. The increased medial PFC activity may have modulated emotional responsiveness in patients via top-down cognitive control, therewith restoring emotional processing at the behavioral level, despite striatal dysfunction. These results may impact upon current treatment strategies of affective disorders in PD as patients may benefit from this intact or even compensatory influence of prefrontal areas when therapeutic strategies are applied that rely on cognitive control to modulate disturbed processing of emotions.

## Introduction

In addition to the characteristic motor symptoms such as tremor, hypokinesia, rigidity, and postural instability, patients suffering from Parkinson’s disease (PD) frequently encounter psychiatric syndromes, such as affective disorders, cognitive deterioration, sleep disturbances, and hallucinations [1]. Several studies have shown that non-motor symptoms in PD can have an even larger impact on the patients’ quality of life and her/his prognosis than the motor symptoms [2–5].

Even in the absence of a psychopathological diagnosis, PD patients may exhibit disturbed emotional processing. Emotional processing refers to conscious and unconscious processes of recognizing, experiencing, and expressing emotions [6]. Recent studies provided conflicting data when examining emotional processing in PD patients. The majority of studies report an intact ability at the behavioral level of PD patients to explicitly recognize and categorize emotions, but an impaired ability to generate autonomic emotional responses [7–14]. However, other studies reported behavioral impairments, mainly for negative emotions [15, 16, 17]. In one of these latter studies, deficits at the behavioral level appeared to be associated with the level of dopamine, although patients were only assessed during their "*OFF*" state [17]. In other studies, patients who were tested both "*ON"* and "*OFF"* dopaminergic medication showed no behavioral deficits compared to healthy controls [7, 9, 10]. Hence the influence of levodopa treatment on emotional processing remains ambiguous. With respect to the neural underpinnings of disturbed emotional processing in PD, some of the studies failed to observe structural or functional deficits [18], while others reported reduced grey matter volume [15, 16], or region-specific alterations of functional activity [7–11, 13, 14, 17, 19–21]. Frontal-subcortical limbic regions including the amygdala, the orbitofrontal cortex (OFC), the ventral anterior cingulate cortex (ACC), the ventral striatum, and the ventrolateral prefrontal cortex (PFC) have been described to be affected in PD. Most of these structures are part of the “emotional circuitry” and depend largely upon dopaminergic projections.

Findings of a relative intact ability in PD patients to consciously recognize and categorize emotions (despite the above mentioned neurobiological alterations) raise the question whether compensational neural mechanisms may be effective in PD. The present study aimed to address this question by investigating (implicit) emotional processing and its neurobiological correlates in 21 medicated PD patients and 21 matched healthy controls (HCs) using event-related blood-oxygen-level dependent fMRI (BOLD-fMRI). We hypothesized that PD patients would show intact explicit emotional processing at the behavioral level. Furthermore, we expected to find evidence for deficient neural processing in striatal and limbic brain areas involved in emotional processing. We further hypothesized that compensatory neural mechanisms during implicit emotional processing in PD may occur, thereby enabling intact emotion recognition abilities at the behavioral level.

## Materials and methods

### Participants

Twenty-one patients with idiopathic PD and 21 matched HCs participated in the study. Patients were recruited from the Movement Disorders Clinic of the Maastricht University Medical Centre (MUMC), the Netherlands. Controls were recruited from a pre-existing database of healthy volunteers generated at the Department of Psychiatry of the MUMC to match the patient group with respect to age, gender, and education. All assessments and scanning sessions took place at the Institute for Neuroscience and Medicine, Research Centre Jülich, Germany.

To be eligible for participation, patients had to fulfill the Queen Square Brain Bank diagnostic criteria for PD [22]. Patients had to be on stable doses of antiparkinsonian medication for at least one month. Patients with neurodegenerative disorders other than PD or controls with neurodegenerative disorders were excluded. For all subjects, the following additional exclusion criteria were applied: major depressive disorder, as defined by the criteria of the fourth edition of the Diagnostic and Statistical Manual (DSM-IV) of the American Psychiatric Association [23], cognitive deterioration operationalized as a score of <26 on the Mini Mental State Examination (MMSE) [24], contra-indications for MRI, and the presence of alcohol- and/or drugs abuse. Written informed consent was obtained prior to participation and according to the guidelines of the Declaration of Helsinki. This study was approved by the ethics committee of the Ärztekammer Nordrhein (2012188).

### Experimental design

#### Stimulus material

In this cross-sectional study, the stimuli consisted of 195 color pictures that were selected from the International Affective Picture System (IAPS) [25] based on their normative valence and arousal ratings. 'Valence' refers to the nature of emotion: positive, neutral or negative, 'arousal' refers to the intensity of emotion, ranging from low to high arousal [26]. The paradigm consisted of three emotional conditions: 65 high arousing negative, 65 high arousing positive, and 65 low arousing neutral pictures. The pictures were balanced with respect to stimulus complexity, colors, presence of human beings and animals. Selected negative pictures included mutilations, threatening animals, human violence etc., with a mean valence rating of 2.3 (SD 0.6) and a mean arousal rating of 6.4 (SD 0.5). Positive pictures included babies, couples, sports activities etc., with a mean valence rating of 7.3 (SD 0.5) and a mean arousal rating of 6.0 (SD 0.6). Neutral pictures included buildings, plants, furniture etc., with a mean valence rating of 5.1 (SD 0.3) and arousal rating of 3.0 (SD 0.4). Mean valence and arousal ratings are based on IAPS normative ratings [25]. Pictures were selected such that negative and positive pictures were significantly more arousing than neutral pictures (*t* = 39.0/31.2 for negative/positive respectively, *P* <0.001). Negative pictures were slightly more arousing than positive pictures (*t* = -4.3, *P* <0.001). For a list of selected IAPS pictures, see S1 Table.

#### Functional MRI paradigm

The experimental task was programmed using Presentation^®^ 16.3 (Neurobehavioral Systems, Inc.). Stimuli were presented on a 30 inch shielded LCD monitor (60 Hz) at a distance of 245 cm and were seen via a mirror system installed on top of the head coil. Vision correction was applied when necessary. Participants were instructed to passively view the pictures during scanning. An event-related design was chosen to avoid habituation and stimulus predictability. In order to avoid long-lasting mood states the pictures were presented in a randomized order, with no more than two pictures of the same valence or arousal category in a row. Thirty ‘null-events’ (i.e., black screen with white fixation cross) were added to the paradigm, leading effectively to variable stimulus-onset asynchronies.

**Fig 1:**
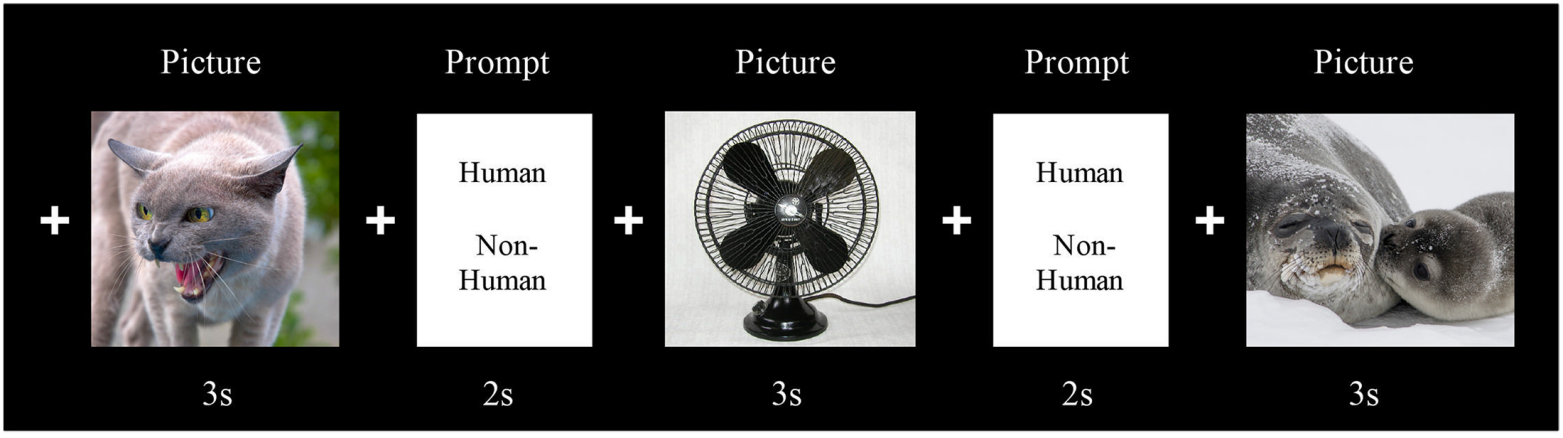
Experimental design of the event-related emotional processing task. Each trial started with a black screen with a white fixation cross, followed by a picture or null-event (fixation cross). After an inter-stimulus interval (fixation cross) a prompt appeared on the screen asking the participants whether the pictured contained a (part of a) human or not. The onsets of both the pictures and prompt were jittered in order to achieve optimal sampling of the hemodynamic response. Note that the pictures depicted in Fig 1 are not from the IAPS. In the actual experiment the words human/non-human were placed next to each other.

Fig 1 represents a schematic illustration of the experimental design. The experiment consisted of one session that lasted for approximately 25 minutes. Each trial started with a black screen with a white fixation cross presented for 2500ms, followed by a picture or null-event presented for 3000ms. After an inter-stimulus interval of 500ms a prompt appeared on the screen for 2000ms asking the participants whether the pictured contained a (part of a) human. They were instructed to answer by pressing with the index and middle finger of their dominant/operating hand corresponding to the left/right position of the ‘human/non-human’ word on the screen. This task was included in order to control for attention without referring to the emotional content of the picture. The error rates for this task were merely used for observatory purposes and not included in the analyses.

The onsets of both the pictures and the prompts were jittered in order to achieve optimal sampling of the hemodynamic response [27]. A practice session was held prior to the fMRI experiment and outside the scanner. The practice task followed the same script as the fMRI task, yet no jittering was added.

#### Post-scanning subjective ratings

Directly after fMRI scanning, participants viewed the same set of pictures in identical order on a computer. They were asked to rate every picture using two independent scales from a paper-and-pencil version of the Self-Assessment Manikin scale [28]. The scales ranged from negative (1) to neutral to positive (9) for valence, and from calm (1) to high arousing (9) for arousal. The post-scanning image ratings were deliberately held outside the scanner, as the emotional evaluation of pictures requires explicit cognitive processes, which may suppress or alter implicit functional emotional reactivity [29, 30].

### fMRI data acquisition

The images were acquired with a 3-T TRIO MRI system (Siemens, Erlangen, Germany) using a T2*-weighted EPI sequence (repetition time = 2200ms, echo time = 30ms). In total, 810 images were acquired, each of which consisted of 36 axial slices with a thickness of 3.1 mm (flip angle = 90°, distance factor = 10%, field of view (FOV) = 200 mm, 64 x 64 matrix resulting in a voxel size of 3.1 × 3.1 × 3.0 mm^2^). The slices covered the whole brain and were acquired parallel to the anterior-posterior commissure line. High-resolution, anatomical images (voxel size 1x1x1 mm^3^) were acquired for anatomical reference using a standard T1-weighted 3D MP-RAGE sequence.

### Behavioral data analysis

Statistical analyses were performed with the Statistical Package for Social Sciences (SPSS, version 21). The chi-square technique was used to compare proportions. Demographic and clinical data were compared with *t*-tests/ANOVA or Mann-Whitney U/ Friedman’s ANOVA test, whenever homogeneity of variances was violated (*P*<0.05; two-tailed).

For the subjective ratings of valence and arousal, within group differences were calculated using paired *t*-tests. Comparisons between groups were performed using multivariate ANOVAs for valence and arousal separately, with picture category (i.e., positive/neutral/negative valence; positive high arousal/neutral low arousal/negative high arousal) as dependent variable, and group as independent or between group variable. Pillai’s Trace *F* approximations are reported. The Bonferroni correction was used to correct for multiple comparisons where necessary.

### fMRI data analysis

The fMRI data were analyzed using the Statistical Parametric Mapping (SPM) Program (Wellcome Department of Imaging Neuroscience, London, United Kingdom www.fil.ion.ucl.ac.uk/spm/software/spm8). The first nine ‘dummy’ images were excluded from analyses. All images were first spatially realigned to correct for inter-scan movement. Then the mean EPI image for each subject was computed and spatially normalized to the Montreal Neurological Institute (MNI) template using the “unified segmentation” function [31]. The data were then smoothed using a Gaussian kernel (full width at half maximum of 8 mm).

Overall, regressors were defined for each picture category (i.e., negative, neutral, positive), indicating the onset times of individual trials (each considered as a single event). For each onset regressor, we included one additional regressor into the design matrix with parametric modulations representing the individual subjective rating of. The hemodynamic response to each event type was modeled using a canonical synthetic hemodynamic response function and its first derivative. The six head movement parameters were included as confounds.

#### Within group comparisons

First-level linear baseline contrasts were calculated comparing each regressor with the implicit baseline (i.e., those time periods that were not explicitly modeled and those during which no event occurred) by setting the regressors of interest to 1 and all other regressors to zero. These contrasts were then taken to the second level where they were subjected to within-subject analysis of variance (ANOVA, flexible factorial design in SPM8) with a single factor condition (positive, neutral, negative) using a family-wise error corrected threshold of *P*_FWE_<0.05 at the voxel-level.

#### Between group comparisons

In order to identify the main effect of the experimental task, first level linear contrasts were calculated, comparing events of the different conditions (e.g., negative>neutral, positive>neutral etc.). These contrasts were taken to the second level where they were subjected to two-sample *t* tests (between group) again using a corrected threshold of *P*_FWE_<0.05 at the voxel-level.

In addition to the analyses at the whole brain level, between group-analyses were performed with a region of interest (ROI) approach. Based on previous imaging studies on emotional processing in PD patients [17] and healthy volunteers [32], four ROIs were identified: the ventrolateral prefrontal cortex (PFC) and bilateral putamen [17] as well as the medial PFC and the amygdala [32]. ROIs were created by computing a spherical volume of interest with a radius of 8mm (corresponding to the Gaussian kernel with a full width at half maximum of 8 mm used for smoothing the single subject data) centered on the previously reported activation peaks of the four ROIs (left ventrolateral PFC: -54, +24, -9; right medial PFC: +3, +54, +27; left putamen: -24, -3, +3; right putamen: +24, +6, -6; left amygdala: -18, 0, -15). For the ROI analyses, significant activations are reported at *P*_SVC_<0.05 (small volume correction, i.e., family-wise error correction within the search volume). Beta values representing estimates of BOLD signal amplitudes were extracted for the maximally activated voxels within the significant clusters revealed by the ROI analyses.

## Results

### Participants

Careful inspection of the imaging data resulted in the exclusion of two PD patients; one due large movement-related artifacts (i.e., >3mm), and one because of a lacunar infarct in the right striatum. The two matching controls were excluded likewise. The remaining study sample thus consisted of 19 PD patients (13 male; mean age = 60.2 ± 9.6 years) and 19 matched HCs (13 male, mean age = 60.8 ± 9.9 years). All patients were assessed in their 'on' state and were taking different combinations of L-dopa (n = 17), L-dopa with COMT inhibitor (n = 1), dopamine agonists (n = 11), MAO inhibitors (n = 5), amantadine (n = 3), and anticholinergic drugs (n = 2). Levodopa equivalent daily doses (LEDD) were calculated in order to control for possible medication effects [33]. None of the controls were taking antidepressants, compared to only one patient, yet in the absence of a major depressive disorder or another current psychiatric disorder.

Table 1 summarizes the demographic and clinical characteristics of the subjects. PD patients and controls did not differ in terms of age, gender, and education, which indicates that groups were well matched. In addition, both groups showed comparable scores for global cognition (MMSE) and executive functioning (Frontal Assessment Battery; FAB) [34]. Compared to controls, PD patients showed significantly higher scores, yet still on a subclinical level, for depression (Hamilton Rating Scale for Depression; HAMD) [35], anxiety (Hamilton Rating Scale for Anxiety; HARS) [36], Parkinson Anxiety Scale total score (PAS) [37], particularly more episodic anxiety (PAS episodic), and apathy (Lille Apathy Rating Scale; LARS) [38]. However, based on the proposed criteria for Apathy [39], five patients (26.3%) could be diagnosed with clinical apathy, compared to zero in the control group. PD patients showed mild to moderate severity of motor symptoms and an average disease duration (post-diagnosis) of 5.3 ± 3.9 years.

**Table 1:** Demographic and clinical characteristics of Parkinson patients and matched healthy controls.

Characteristics	Parkinson (*n* = 19)	Controls (*n* = 19)	*P*-value
Age	60.2 (9.6)	60.8 (9.9)	0.86
Gender (% male)	68.4%	68.4%	1.00
Education (years)	13.2 (2.7)	13.95 (4.0)	0.48
Handedness (right)	17	17	1.00
HAM-D	5.2 (4.5)	2.5 (2.6)	0.03*
HARS	5.7 (4.3)	2.2 (2.2)	0.01*
PAS			
*Total score*	8.3 (6.7)	4.3 (4.7)	0.04*
*Persistent*	5.4 (4.4)	3.3 (3.5)	0.11
*Episodic*	1.5 (1.8)	0.5 (1.1)	0.04*
*Avoidance*	1.4 (2.1)	0.5 (1.0)	0.19
MMSE	29.1 (1.2)	29.4 (0.8)	0.35
FAB	16.8 (1.3)	16.5 (1.3)	0.55
LARS	- 23.6 (7.7)	- 28.7 (5.1)	0.04*
Apathy Criteria (% yes)	26.3%	0.0%	0.02*
UPDRS II	12.0 (5.3)	--	--
UPDRS III	23.8 (8.6)	--	--
UPDRS IV	2.3 (2.2)	--	--
Schwab & England	84.7 (7.4)	--	--
Hoehn-Yahr (median)	2.5 (0.5; range 1–3)	--	--
LEDD (mg/day)	448.4 (213.6)	--	--

Note: Means and standard deviations, unless otherwise indicated. HAMD, Hamilton Rating Scale for Depression; HARS, Hamilton Anxiety Rating Scale; PAS, Parkinson Anxiety Scale; MMSE, Mini Mental State Examination; FAB, Frontal Assessment Battery; LARS, Lille Apathy Rating Scale; UPDRS, Unified Parkinson’s Disease Rating Scale (section II-IV); LEDD, levodopa equivalent daily dose.

* Significant group differences at *P* < .05.

### Behavioral results

Valence and arousal ratings for PD patients and controls are presented in Table 2. In both groups, positive/negative pictures were considered to be more positive/negative (PD: *t*(18) = 15.38/-21.74, *P*<0.001; HCs: *t*(18) = 12.76/-18.15, *P*<0.001) and more arousing (PD: *t*(18) = 18.25/13.13, *P*<0.001; HCs: *t*(18) = 8.33/9.29, *P*<0.001) than neutral pictures. Also, both groups considered negative pictures to be more negative (PD: *t*(18) = -21.12, *P*<0.001; HCs: *t*(18) = -17.43, *P*<0.001) and more arousing (PD: *t*(18) = 8.42, *P*<0.001; HCs: *t*(18) = 3.66, *P*<0.001) than positive pictures. All pairwise comparisons were corrected for multiple comparisons (Bonferroni). PD patients and HCs did not show significant between group differences for both valence and arousal ratings.

**Table 2:** Subject valence and arousal ratings for Parkinson patients (n = 19) and healthy controls (n = 19).

	Positive/high	Neutral/low	Negative/high	*P-* value
*Valence ratings*				
PD	6.8 (0.7)	5.3 (0.3)	2.2 (0.4)	<0.001*
Controls	7.0 (0.8)	5.4 (0.4)	2.2 (0.6)	<0.001*
PD vs. Controls	ns	ns	ns	
*Arousal ratings*				
PD	4.7 (1.2)	2.1 (0.8)	6.9 (1.1)	<0.001*
Controls	4.6 (1.7)	2.1 (1.1)	6.1 (1.6)	<0.001*
PD vs. Controls	ns	ns	ns	

Note: Depicted are means and standard deviations. The 0–9 SAM Scale was used: valence ratings, 0 is most negative and 9 is most positive; arousal ratings, 0 is lowest arousal and 9 is highest arousal. ns: no significant group difference.

*Significant within-group differences between conditions.

### BOLD fMRI results

#### Within group differences

Multiple significant within group differences of activated brain regions and structures were found in PD patients and HCs for the different emotional categories (all *P*_FWE_<0.05, see S2 and S3 Tables). Here, we only present within group differences when comparing pictures depicting high levels of arousal (i.e., positive and negative collapsed) with those depicting low levels of arousal (i.e., high>low). PD patients and controls both showed increased activity in the left and right middle temporal gyrus (MTG), left and right fusiform gyrus, left and right middle occipital gyrus (MOG), left OFC, and left and right ventrolateral PFC (vlPFC). However, controls showed more pronounced activity in the left thalamus and the brainstem, while PD patients showed more pronounced activity in the right OFC, and left and right dorsomedial PFC (dmPFC). For the opposite contrast (i.e., low>high), controls only showed increased activity in the left fusiform gyrus.

#### Between group differences

At the predefined whole brain threshold of *P*_FWE_<0.05 (at the voxel-level), there were no significant between-group differences. However, Table 3 summarizes the significant differences in brain activation between PD patients and controls for the modulation of valence and arousal as revealed by the ROI-analyses. Note that the ROI analyses for the ventrolateral PFC and the amygdala did not reveal any significant results, hence results are not shown.

**Table 3:** Functional differences between Parkinson patients (PD) and healthy controls (HC) as revealed by region-of-interest analyses (ROI).

Contrasts	Brain region	Cluster size	MNI Coordinates x/y/z			T-value	*P*-value
Valence							
Positive							
HC > PD	L Putamen	21	-24	-6	10	3.81	0.010
PD > HC	R Dorsomedial prefrontal cortex	177	4	62	20	5.25	0.002
Arousal							
Low arousal							
HC > PD	L Putamen	201	-20	10	-2	4.23	0.018
PD > HC	R Dorsomedial prefrontal cortex	75	8	62	24	4.10	0.022
High Arousal							
HC > PD	L Putamen	131	-24	-6	10	4.49	0.002
	R Putamen	122	24	10	2	4.06	0.008
PD > HC	R Dorsomedial prefrontal cortex	104	8	64	26	4.63	0.009

Cluster size denotes the extent of the activation cluster by number of significant voxels (k_E_). MNI coordinates refer to the location of the maximally activated voxel (peak) within an activation cluster. Results are considered significant at P_SVC_ < .05 (FWE-corrected for small volume/ region-of-interest).

Note: high arousal means positive and negative pictures collapsed. Low arousing stimuli are equivalent to neutral pictures

**Fig 2:**
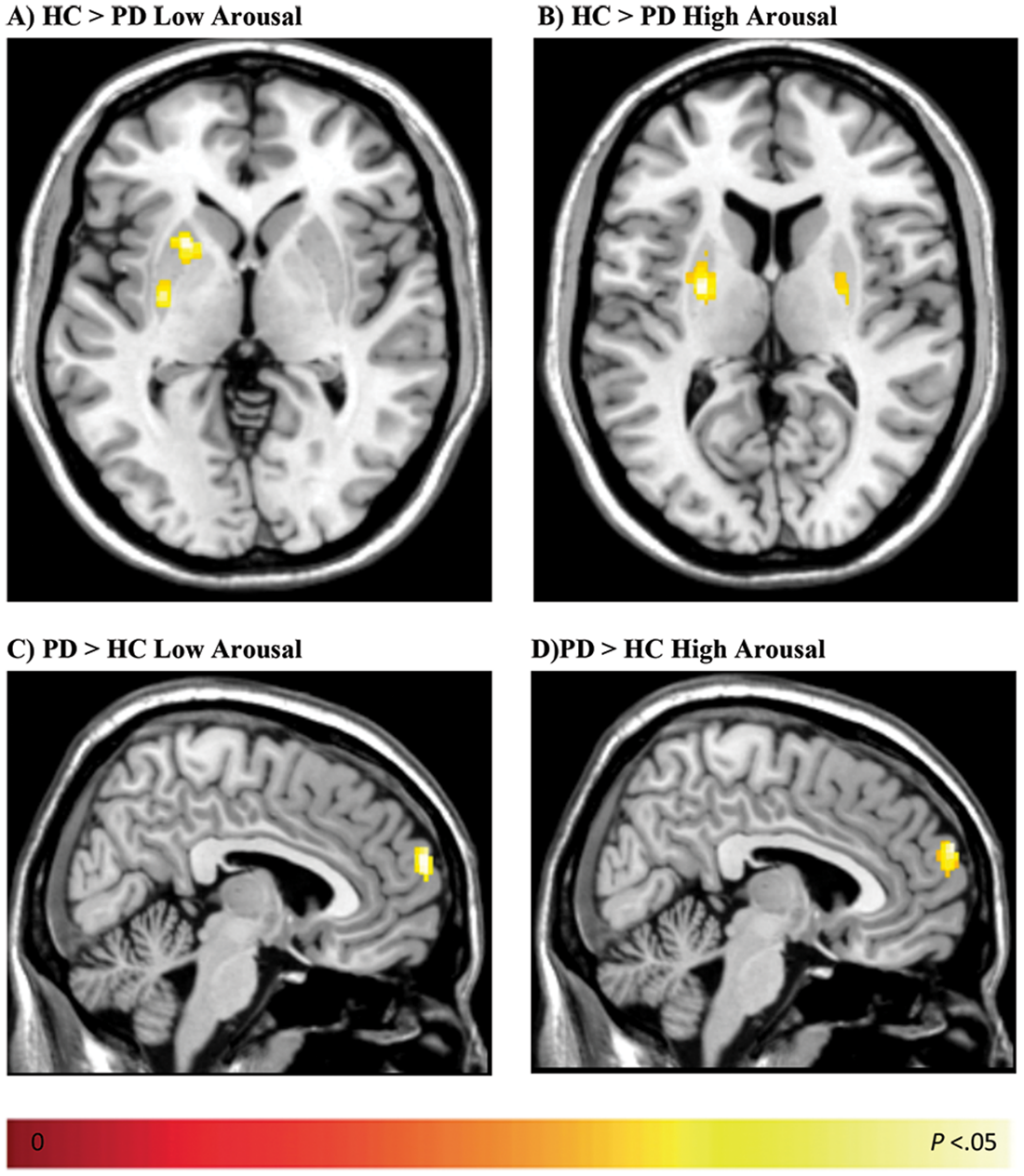
Results of the ROI analysis on the processing of low and high arousing stimuli in Parkinson patients and controls. Contrasting healthy controls (HCs) with Parkinson patients (PD) showed a differential increase of BOLD signal for HCs in the left (posterior) putamen for low arousal (**A**) and bilaterally in the putamen for high arousal (**B**, all *P*_SVC_<0.05). Contrasting Parkinson patients with healthy controls revealed an increased BOLD signal for Parkinson patients in the right dorsomedial prefrontal cortex (dmPFC) for low (C) arousal and high arousal (D, all *P*_SVC_<0.05).

For stimuli with positive valence, PD patients (compared to controls) showed increased activity in the right dmPFC, while controls (compared to patients) showed increased activity in the left putamen (both *P*_SVC_<0.05, see Table 3). For arousal, negative and positive pictures were collapsed as high arousing pictures, while neutral pictures served as low arousing pictures, based on the individual subjective arousal ratings. During the (implicit) processing of both low and high arousing pictures, controls showed differential activity in the left (and right) posterior putamen compared to PD patients (*P*_SVC_<0.05, see Fig 2 and Table 3). However, close inspection of the activated clusters (i.e., the BOLD signal changes as indexed by the beta values of the most active voxels) revealed that left (posterior) putamen activity was reduced in patients, as reflected by the negative beta values. Moreover, although the reduced activity was found for both low and high arousing stimuli, it appeared to be most pronounced for high arousing stimuli (S1 Fig).

In contrast to controls, PD patients showed differential activity in the right dmPFC (*P*_SVC_<0.05, see Fig 2 and Table 3) when processing low and high arousing pictures. The beta values of the most active voxels within the dmPFC clusters revealed increased right dmPFC activity in PD patients, but also a pronounced decrease of right dmPFC activity in controls, again most prominent for the high arousing stimuli (S2 Fig).

All between group differences remained significant after correction for symptoms of apathy, depression, anxiety, and use of antidepressants. Functional activity in patients was also not significantly influenced by levodopa usage, disease duration, and severity of motor symptoms.

#### Correlation analyses of BOLD fMRI data with behavioral and clinical data

Table 4 presents the significant correlations between clinical scores, task performance, and the BOLD signal in the maximally activated voxels within the significant clusters. Note that negative mean beta values should be interpreted as less activity and positive mean beta values as increased activity.

**Table 4:** Significant correlations between clinical scores, performance, and the BOLD signal in the maximally activated voxels in the significant clusters/ROIs.

L post putamen			R post putamen	R dmPFC		
	Low	Positive	High	High	Low	Positive
*PD patients*	*ß = -0*.*32*	*ß = -0*.*63*	*ß = -1*.*19*	*ß = 1*.*61*	*ß = -0*.*19*	*ß = 0*.*55*
PAS total score	-	-	-	-0.65/<0.01	-	-
PAS persistent	-	-	-	-0.58/<0.01	-	-
PAS episodic	-	-	-	-0.72/<0.01	-0.47/0.04	-
PAS avoidance	0.52/0.02	0.53/0.02	0.48/0.04	-	-	-
LARS (dichotomized)	-	-	-	-0.47/0.04	-	-
Neutral valence ratings	-	-	-	0.61/<0.01	-	-
Positive valence ratings	-	-	-	0.57/<0.01	-	-
Low arousal ratings	-	-	-	-	-	0.50/0.03
*Controls*	*ß = 0*.*48*	*ß = 0*.*02*				
MMSE	-0.62/<0.01	-	-	-	-	-
PAS episodic	-	-0.56/0.01	-	-	-	-
FAB	-	-0.63/ < .01	-	-	-	-

Correlation coefficients refer to Pearson’s correlation or Spearman’s rank correlation, depending on normality (*r*/*P*-value). Low/High/Positive denotes the level or category of arousal. *ß*, mean beta value; dmPFC, dorsomedial prefrontal cortex; FAB, Frontal Assessment Battery; LARS, Lille Apathy Rating Scale; MMSE, Mini Mental State Examination; PAS, Parkinson Anxiety Scale; PD, Parkinson; Post, posterior.

## Discussion

We investigated the neurobiology of emotional processing in Parkinson’s disease (PD) with event-related BOLD-fMRI in patients with mild to moderate PD and matched healthy controls (HCs) during a standardized implicit emotional processing task. PD patients were capable of explicitly discriminating and rating the intensity of emotions, as shown by similar ratings as controls. However, PD patients showed reduced functional activity of the left (and right) posterior putamen, and an increase of activity in the right dmPFC, both most pronounced in response to high arousing emotional stimuli. These results are in line with prior neuroimaging studies in PD that showed an intact ability to recognize and categorize emotions in the presence of frontal-subcortical limbic circuitry abnormalities [7, 8, 10]. Importantly, our findings extend previous work by revealing for the first time a possible compensatory neural mechanism in PD patients, with increased medial frontal activity that may have compensated for striatal dysfunction during emotion processing.

The reduced (bilateral) posterior putaminal activity in PD patients can probably be related to the pathology of PD. Several studies have shown that putaminal volume is significantly reduced in PD patients, and exhibits increasing atrophy as the disease progresses [40, 41]. Resting state functional connectivity research showed reduced correlations of striatal activity with activity in the thalamus, midbrain, pons, and brainstem in PD patients, most pronounced for the posterior putamen [42]. Also, loss of dopaminergic neurons in the substantia nigra subsequently results in reduced striatal dopamine transporter availability (DAT), amongst others in the putamen [17]. Although the putamen, and the striatum in general, is traditionally considered to be associated with motor function, there is growing evidence emphasizing its role in the modulation of (emotional) behavior and cognition. Being part of several cortico-subcortical loops, its direct input from and projections to (pre)frontal areas via interconnectivity between striatal regions and/or through its connections with the globus pallidus and substantia nigra form a crucial linkage between motor and emotional regions of the brain [43, 44]. Moreover, Phillips and colleagues proposed a neurobiological framework of emotional processing where the striatum (including the putamen) has strong connections with an affective neurocircuitry that is particularly involved in the implicit perception of emotional stimuli and the generation of a physiological response [6]. Considering the critical role of intact dopaminergic neurotransmission within this network [45], reduced putaminal DAT may disturb emotional reactivity remarkably.

Increased involvement of prefrontal structures on the other hand, is a common phenomenon in the elderly. According to the compensation-related utilization of neural circuits hypothesis (CRUNCH) [46], older adults’ brains are capable of addressing alternative neural resources, such as the PFC, in order to compensate for functional decline elsewhere in the brain. The same principle of compensational neural activity may be applicable to functional decline due to neurodegenerative diseases. Findings on functional connectivity between prefrontal and subcortical regions support the view of a direct excitatory influence of the dmPFC on both amygdalar and autonomic brain regions [47–49]. Furthermore, the medial prefrontal network (MPN) is known to be highly involved in the interaction between cognitive and emotional processes in the brain [50] as it can modulate (bottom-up) automatic processes arising from activated subcortical limbic regions via top-down cognitive control in order to regulate our emotions [6, 51, 52]. Despite disturbed subcortical activity (here: putaminal) in our PD sample, patients were unimpaired in rating the emotional value and intensity of pictures, which requires prefrontal cognitive control processes such as context processing and decision-making. In addition, we found that increased right dmPFC activity for intense emotional stimuli was associated with both lower levels of anxiety and lower levels of apathy in PD patients, which may provide another indication that the dmPFC is able the control subcortical limbic functioning (Table 4). As such, the increased right dmPFC activity in our PD sample may have served as a (top-down) cognitive control mechanism, compensating for the disturbed subcortical activity and enabling normal emotion regulation in patients.

An imbalance in the emotional system as reflected by either impaired cortical cognitive control or overactive subcortical bottom-up processes may contribute to several psychiatric disorders such as depression or anxiety [53]. As such, medial PFC functioning may be an important target when treating affective disorders. Recent functional imaging studies in patients without PD have shown that nonpharmacological treatments such as Cognitive Behavioral Therapy (CBT) can restore impaired neuronal affective processing by increasing functional connectivity between limbic and prefrontal cortices [54, 55]. Hence, PD patients who are cognitively intact may take advantage of the preserved and potential compensatory involvement of medial prefrontal cortices in emotion regulation, despite disturbed subcortical activity related to the pathology of PD. Therapeutic interventions, such as CBT, that rely on frontal cognitive control mechanisms or emotion regulation strategies may therefore be particularly useful when treating affective disorders in this specific population.

The following limitations of the present study need to be considered. All patients (except for one) were assessed in their 'on' state and were taking stable doses of antiparkinsonian medication. As dopamine replacement therapy (DRT) can partially restore the loss of dopamine, and therewith improve functional reactivity [7, 10], studying patients off medication seems preferable in order to study the unmasked effects of the disease and to see whether depletion of dopamine can be associated with emotional processing deficits even at the behavioral level. However, in unmedicated PD patients, motor symptoms like tremor or dystonia can cause movement artifacts that may confound neuroimaging findings. Moreover, despite receiving DRT the patients in our sample still showed significant motor impairments (as assessed with the UPDRS-III), which indicates that the DRT did not fully compensate for the loss of dopamine. On the other hand, increasing levodopa dose in order to further improve motor symptoms may lead to overstimulation of otherwise unaffected (limbic) regions which in turn could counteract non-motor processes such as emotional reactivity [10]. In spite of this, the differential neural activation patterns in PD patients as revealed by our study were statistically robust against the effects of levodopa dose and severity of motor symptoms as well as disease duration. Future studies should ideally test patients on *and* off medication, in order to study the influence of dopamine depletion, or even overstimulation, on emotional processing. Another putative limitation concerns the use of a set of emotional and neutral stimuli (i.e., IAPS) that were normalized in a non-European sample, whereas all of our participants were of European origin and may have different standards when it comes to rating a picture as negative or positive. This may explain the similar trends for high and low arousing stimuli shown by our fMRI results, since both PD patients and HCs tended to evaluate neutral pictures as slightly more positive compared to the normative IAPS ratings. Nonetheless, by calculating the beta values for each arousal category, we were able to reveal that the differences in functional activity between patients and controls were most pronounced for high arousing stimuli. Finally, our additional attention check in which participants had to decide whether or not they saw a human in the picture may have elicited some frontal cognitive activation related to decision-making, and therewith possibly suppressed limbic area activity for instance in the amygdala. This, however, seems unlikely as most limbic regions were equally active in both patients and controls, except for the posterior putamen.

In conclusion, our data on emotional processing showed decreased striatal reactivity in PD patients in response to intense emotional stimuli, without any deficit in explicitly evaluating and rating the intensity of emotions compared to matched healthy controls. We further observed increased prefrontal activation in right dorsomedial regions that may have served as a compensatory top-down cognitive control mechanism by restoring dysfunction in subcortical limbic circuitry related to the pathology of the disease and therewith modulating emotion regulation in PD patients. PD patients who are cognitively intact could possibly benefit from the intact or even compensatory influence of prefrontal areas in the therapeutic treatment of affective disorders.

## Supporting information

S1 Table Pictures from the International Affective Picture System (IAPS) included in the present study. The numbers refer to the pictures included in this study.
(DOCX)

S2 Table Within group differences for Parkinson patients (n = 19). Cluster size denotes the extent of the activation cluster by number of significant voxels (k_E_). MNI coordinates refer to the location of the maximally activated voxel (peak) within an activation cluster. Results are considered significant at *P*<0.05 (FWE corrected at the peak/voxel level).
(DOCX)

S3 Table Within group differences for healthy controls (n = 19). Cluster size denotes the extent of the activation cluster by number of significant voxels (k_E_). MNI coordinates refer to the location of the maximally activated voxel (peak) within an activation cluster. Results are considered significant at *P*<0.05 (FWE corrected at the peak/voxel level).
(DOCX)

S1 Fig Mean beta values derived from the ROI analysis for the left posterior putamen in Parkinson patients (PD) and healthy controls (HC). MNI coordinates refer to the location of the maxima. Results are considered significant at *P*_FWE_<0.05 small volume correction.
(DOCX)

S2 Fig Mean beta values of ROI analyses right dorsomedial prefrontal cortex in Parkinson patients (PD) and healthy controls (HC). MNI coordinates refer to the location of the maxima. Results are considered significant at *P*_FWE_<0.05 small volume correction.
(DOCX)
