# Supplementary material for: An fMRI study into emotional processing in Parkinson’s disease: Does increased medial prefrontal activation compensate for striatal dysfunction?
Source: PLoS One. 2017 May 9;12(5):e0177085. doi: 10.1371/journal.pone.0177085 (PMC5423613; doi:10.1371/journal.pone.0177085)
Supplement: S1 Table — The numbers refer to the pictures included in this study. (DOCX) [file pone.0177085.s001.docx]

**S1 Table.**

| **Neutral** | **Positive** | **Negative** |
| --- | --- | --- |
| 2002 | 1650 | 1120 |
| 2102 | 1710 | 1525 |
| 2191 | 1811 | 1930 |
| 2214 | 2045 | 2053 |
| 2215 | 2058 | 2095 |
| 2273 | 2071 | 2345.1 |
| 2357 | 2075 | 2683 |
| 2377 | 2216 | 2688 |
| 2382 | 2345 | 2691 |
| 2383 | 2347 | 2692 |
| 2393 | 4220 | 2703 |
| 2446 | 4520 | 2800 |
| 2518 | 4597 | 2811 |
| 2570 | 4598 | 2981 |
| 2745.1 | 4599 | 3001 |
| 2880 | 4607 | 3030 |
| 2890 | 4608 | 3053 |
| 5040 | 4623 | 3060 |
| 5130 | 4626 | 3071 |
| 5390 | 4660 | 3102 |
| 5471 | 4676 | 3103 |
| 5520 | 5260 | 3110 |
| 5731 | 5450 | 3170 |
| 5740 | 5470 | 3195 |
| 6150 | 5480 | 3266 |
| 7001 | 5621 | 3350 |
| 7002 | 5623 | 3400 |
| 7003 | 5626 | 3500 |
| 7004 | 5629 | 6021 |
| 7009 | 5700 | 6190 |
| 7012 | 5825 | 6212 |
| 7014 | 5833 | 6231 |
| 7017 | 7220 | 6243 |
| 7020 | 7230 | 6260 |
| 7025 | 7270 | 6300 |
| 7026 | 7330 | 6312 |
| 7032 | 7405 | 6315 |
| 7034 | 7451 | 6540 |
| 7035 | 7502 | 6550 |
| 7036 | 7650 | 6560 |
| 7040 | 8030 | 6821 |
| 7041 | 8034 | 6830 |
| 7045 | 8080 | 9075 |
| 7050 | 8090 | 9163 |
| 7052 | 8158 | 9183 |
| 7053 | 8161 | 9250 |
| 7056 | 8163 | 9252 |
| 7059 | 8170 | 9254 |
| 7060 | 8178 | 9332 |
| 7080 | 8179 | 9410 |
| 7100 | 8180 | 9412 |
| 7140 | 8185 | 9413 |
| 7150 | 8186 | 9414 |
| 7175 | 8190 | 9424 |
| 7179 | 8210 | 9433 |
| 7190 | 8370 | 9495 |
| 7205 | 8380 | 9570 |
| 7217 | 8420 | 9600 |
| 7290 | 8470 | 9622 |
| 7491 | 8490 | 9635.1 |
| 7547 | 8496 | 9810 |
| 7705 | 8499 | 9902 |
| 7710 | 8501 | 9908 |
| 7950 | 8502 | 9921 |
| 8312 | 8531 | 9940 |
