# Supplementary material for: An fMRI study into emotional processing in Parkinson’s disease: Does increased medial prefrontal activation compensate for striatal dysfunction?
Source: PLoS One. 2017 May 9;12(5):e0177085. doi: 10.1371/journal.pone.0177085 (PMC5423613; doi:10.1371/journal.pone.0177085)
Supplement: S2 Table — Cluster size denotes the extent of the activation cluster by number of significant voxels (kE). MNI coordinates refer to the location of the maximally activated voxel (peak) within an activation cluster. Results are considered significant at P<0.05 (FWE corrected at the peak/voxel level). (DOCX) [file pone.0177085.s002.docx]

**S2 Table.**

| **Contrasts** | **Cluster size (k_E_)** | | **MNI Coordinates x/y/z** | | | **T-value** | ***P*-value** |
| --- | --- | --- | --- | --- | --- | --- | --- |
| **VALENCE** | |  |  |  |  |  |  |
| **Positive > Neutral** | |  |  |  |  |  |  |
| R Middle temporal gyrus | | 4851 | 52 | -54 | 4 | 8.87 | .000 |
| R Fusiform gyrus (posterior) | |  | 46 | -48 | -26 | 8.78 | .000 |
| R Inferior temporal/occipital gyrus | |  | 50 | -74 | -4 | 8.62 | .000 |
| L Middle occipital gyrus | | 3540 | -50 | -80 | 8 | 8.23 | .000 |
| L Middle temporal gyrus (posterior) | |  | -62 | -58 | 6 | 5.94 | .019 |
| L Middle temporal gyrus | |  | -56 | -68 | 6 | 5.82 | .026 |
| **Neutral > Positive** | |  |  |  |  |  |  |
| No significant difference | |  |  |  |  |  |  |
| **Positive >Negative** | |  |  |  |  |  |  |
| R Precentral gyrus | | 5706 | 20 | -18 | 64 | 5.78 | .028 |
|  | |  | 14 | -32 | 56 | 5.71 | .034 |
| R Superior frontal gyrus | |  | 14 | -2 | 60 | 5.70 | .034 |
| **Negative > Positive** | |  |  |  |  |  |  |
| R Lateral Occipital cortex | | 1366 | 46 | -72 | -6 | 5.90 | .021 |
| R Fusiform gyrus (posterior) | |  | 44 | -46 | -24 | 5.71 | .034 |
| L Lateral Occipital cortex | | 1249 | -40 | -80 | -6 | 6.89 | .002 |
| L Fusiform gyrus (posterior) | |  | -42 | -48 | -20 | 6.75 | .002 |
| R Ventrolateral prefrontal cortex | | 1039 | 52 | 30 | 10 | 6.88 | .002 |
| R Ventrolateral prefrontal cortex | |  | 46 | 18 | 26 | 6.59 | .003 |
| R Precentral gyrus | |  | 38 | 10 | 26 | 5.60 | .044 |
| **Neutral > Negative** | |  |  |  |  |  |  |
| L Precentral gyrus | | 4034 | -10 | -20 | 56 | 6.27 | .008 |
| L Postcentral gyrus | |  | -44 | -26 | 58 | 6.09 | .013 |
| L Precentral gyrus | |  | -28 | -16 | 52 | 5.61 | .044 |
| R Superior temporal gyrus | | 2217 | 54 | 2 | 2 | 5.91 | .020 |
| R Superior temporal gyrus (posterior) | |  | 62 | -22 | 0 | 5.84 | .025 |
| L Superior temporal gyrus | | 2008 | -48 | -4 | 8 | 5.89 | .022 |
| L Superior temporal gyrus (posterior) | |  | -48 | -32 | 12 | 5.81 | .026 |
| L Planum temporale | |  | -66 | -12 | 0 | 5.73 | .032 |
| R Inferior parietal lobule | | 519 | 60 | -58 | 40 | 6.25 | .008 |
| R Hippocampus | | 321 | 22 | -42 | 10 | 5.56 | .049 |
| **Negative > Neutral** | |  |  |  |  |  |  |
| L Middle occipital gyrus | | 7620 | -50 | -78 | 8 | 12.59 | .000 |
| L Fusiform gyrus | |  | -42 | -48 | -20 | 12.27 | .000 |
| L Inferior occipital gyrus | |  | -40 | -80 | -6 | 11.68 | .000 |
| R Fusiform gyrus | | 7040 | 44 | -48 | -24 | 14.38 | .000 |
| R Inferior occipital gyrus | |  | 50 | -74 | -4 | 13.77 | .000 |
| R Middle temporal gyrus | |  | 54 | -54 | 4 | 13.43 | .000 |

**S2 Table (continued).**

| **Contrasts** | **Cluster size (k_E_)** | **MNI Coordinates x/y/z** | | | **T-value** | ***P*-value** |
| --- | --- | --- | --- | --- | --- | --- |
| **VALENCE** |  |  |  |  |  |  |
| **Negative > Neutral (continued)** |  |  |  |  |  |  |
| L Ventrolateral prefrontal cortex | 3209 | -44 | 22 | 18 | 7.16 | .001 |
| L Dorsomedial prefrontal cortex |  | -42 | 16 | 24 | 7.12 | .001 |
| L Orbitofrontal cortex |  | -28 | 32 | -20 | 6.35 | .006 |
| R Dorsomedial prefrontal cortex | 3006 | 46 | 18 | 26 | 10.62 | .000 |
| R Ventrolateral prefrontal cortex |  | 52 | 32 | 10 | 8.43 | .000 |
| R Inferior frontal gyrus |  | 44 | 32 | 8 | 8.07 | .000 |
| R Superior dorsomedial prefrontal cortex | 2700 | 6 | 52 | 22 | 7.87 | .000 |
| R Superior dorsomedial prefrontal cortex |  | 4 | 50 | 30 | 6.89 | .002 |
| R Posterior cingulate gyrus | 1147 | 2 | -52 | 24 | 6.18 | .010 |
| L Superior parietal lobule | 706 | -24 | -54 | 44 | 7.52 | .000 |
| R Superior parietal lobule | 649 | 28 | -50 | 48 | 8.43 | .000 |
| R Orbitofrontal cortex | 287 | 4 | 48 | -24 | 5.95 | .018 |
| R Middle temporal gyrus | 229 | 54 | 0 | -24 | 6.17 | .011 |
| R Brainstem | 202 | 8 | -28 | -8 | 5.68 | .037 |
| **AROUSAL** |  |  |  |  |  |  |
| **High Arousal > low arousal** |  |  |  |  |  |  |
| L Middle occipital gyrus | 7351 | -50 | -78 | 8 | 12.00 | .000 |
| L Fusiform gyrus |  | -42 | -48 | -20 | 10.27 | .000 |
| L Inferior occipital gyrus |  | -42 | -80 | -4 | 9.63 | .000 |
| R Fusiform gyrus | 7034 | 44 | -48 | -24 | 13.34 | .000 |
| R Inferior occipital gyrus |  | 50 | -74 | -4 | 12.93 | .000 |
| R Middle temporal gyrus |  | 54 | -54 | 4 | 12.85 | .000 |
| R Posterior cingulate gyrus | 1789 | 2 | -52 | 24 | 5.92 | .020 |
| R Dorsomedial prefrontal cortex | 1752 | 44 | 18 | 26 | 8.55 | .000 |
| R Inferior frontal gyrus |  | 44 | 32 | 8 | 6.28 | .008 |
| R Ventrolateral prefrontal cortex |  | 52 | 32 | 10 | 5.85 | .024 |
| R Superior dorsomedial prefrontal cortex | 1677 | 6 | 56 | 20 | 6.22 | .009 |
| L Dorsomedial prefrontal cortex | 1240 | -42 | 16 | 24 | 6.12 | .012 |
| L Ventrolateral prefrontal cortex |  | -44 | 22 | 18 | 5.67 | .038 |
| L Superior parietal lobule | 817 | -24 | -54 | 44 | 7.03 | .001 |
| R Superior parietal lobule | 710 | 28 | -52 | 50 | 6.94 | .001 |
| R Orbitofrontal cortex | 387 | 4 | 48 | -22 | 5.67 | .038 |
| **Low arousal > high arousal** |  |  |  |  |  |  |
| No significant difference |  |  |  |  |  |  |
