# Supplementary material for: An fMRI study into emotional processing in Parkinson’s disease: Does increased medial prefrontal activation compensate for striatal dysfunction?
Source: PLoS One. 2017 May 9;12(5):e0177085. doi: 10.1371/journal.pone.0177085 (PMC5423613; doi:10.1371/journal.pone.0177085)
Supplement: S3 Table — Cluster size denotes the extent of the activation cluster by number of significant voxels (kE). MNI coordinates refer to the location of the maximally activated voxel (peak) within an activation cluster. Results are considered significant at P<0.05 (FWE corrected at the peak/voxel level). (DOCX) [file pone.0177085.s003.docx]

**S3 Table.**

| **Contrasts** | **Cluster size (k_E_)** | **MNI Coordinates x/y/z** | | | **T-value** | ***P*-value** |
| --- | --- | --- | --- | --- | --- | --- |
| **VALENCE** |  |  |  |  |  |  |
| **Positive > Neutral** |  |  |  |  |  |  |
| R Middle temporal gyrus | 3502 | 56 | -66 | 4 | 12.85 | .000 |
| R Fusiform gyrus |  | 44 | -44 | -22 | 7.18 | .025 |
| R Middle occipital/temporal gyrus |  | 40 | -72 | 20 | 6.24 | .006 |
| L Middle occipital gyrus | 2421 | -50 | -76 | 4 | 11.66 | .000 |
| L Fusiform gyrus |  | -40 | -70 | -12 | 6.45 | .004 |
| L Middle temporal gyrus |  | -50 | -62 | 8 | 6.31 | .005 |
| **Neutral > Positive** |  |  |  |  |  |  |
| No significant difference |  |  |  |  |  |  |
| **Positive >Negative** |  |  |  |  |  |  |
| No significant difference |  |  |  |  |  |  |
| **Negative > Positive** |  |  |  |  |  |  |
| R Middle temporal gyrus | 2368 | 56 | -66 | 4 | 8.33 | .000 |
| R Inferior temporal gyrus |  | 46 | -52 | -18 | 6.91 | .001 |
| R Fusiform gyrus |  | 44 | -60 | -14 | 6.63 | .002 |
| L Inferior occipital gyrus | 1965 | -50 | -78 | 4 | 7.00 | .001 |
| L Fusiform gyrus |  | -42 | -68 | -12 | 6.16 | .008 |
| L Middle temporal gyrus |  | -52 | -60 | 6 | 5.70 | .026 |
| R Superior frontal gyrus | 978 | 8 | 56 | 40 | 6.17 | .008 |
| R Ventrolateral prefrontal cortex | 678 | 56 | 30 | 14 | 8.33 | .000 |
| **Neutral > Negative** |  |  |  |  |  |  |
| L Caudate Nucleus | 718 | -26 | -10 | 28 | 5.69 | .027 |
| L Parahippocampal gyrus | 681 | -34 | -50 | 0 | 5.58 | .000 |
| L Postcentral gyrus | 511 | -46 | -28 | 60 | 6.02 | .011 |
| R Inferior parietal lobule | 291 | 54 | -64 | 42 | 5.81 | .020 |
| **Negative > Neutral** |  |  |  |  |  |  |
| R Middle temporal gyrus | 6599 | 56 | -66 | 4 | 21.18 | .000 |
| R Fusiform gyrus |  | 44 | -50 | -18 | 13.30 | .000 |
| L Middle occipital gyrus | 6186 | -50 | -76 | 4 | 18.40 | .000 |
| L Fusiform gyrus |  | -40 | -70 | -12 | 12.48 | .000 |
| L Middle temporal gyrus |  | -50 | -60 | 8 | 11.97 | .000 |
| R Superior frontal gyrus | 3532 | 6 | 50 | 30 | 7.19 | .001 |
| R Superior medial frontal gyrus |  | 8 | 44 | 40 | 6.64 | .002 |
| L Superior frontal gyrus |  | -6 | 48 | 34 | 6.44 | .004 |
| L Orbitofrontal cortex | 2069 | -40 | 24 | -20 | 9.50 | .000 |
| L Ventrolateral prefrontal cortex |  | -52 | 34 | 4 | 7.47 | .000 |
| R Ventrolateral prefrontal cortex | 1732 | 54 | 32 | 8 | 11.75 | .000 |
| R Inferior frontal gyrus |  | 42 | 14 | 24 | 8.85 | .000 |

**S3 Table (continued).**

| **Contrasts** | **Cluster size (k_E_)** | **MNI Coordinates x/y/z** | | | **T-value** | ***P*-value** |
| --- | --- | --- | --- | --- | --- | --- |
| **VALENCE** |  |  |  |  |  |  |
| **Negative > Neutral (continued)** |  |  |  |  |  |  |
| R Posterior cingulate gyrus | 1723 | 2 | -52 | 30 | 7.04 | .001 |
| R Posterior parietal lobule |  | 28 | -52 | 46 | 5.87 | .017 |
| R Amygdala |  | 16 | -2 | -22 | 5.55 | .038 |
| Orbitofrontal cortex | 363 | 0 | 56 | -20 | 6.03 | .011 |
| L Orbitofrontal cortex |  | -2 | 48 | -22 | 5.72 | .024 |
| **AROUSAL** |  |  |  |  |  |  |
| **High Arousal > Low arousal** |  |  |  |  |  |  |
| R Middle temporal gyrus | 5984 | 56 | -66 | 4 | 19.65 | .000 |
| R Fusiform gyrus |  | 44 | -50 | -20 | 11.61 | .000 |
| R Middle occipital/temporal gyrus |  | 40 | -70 | 20 | 8.48 | .000 |
| L Middle occipital gyrus | 5621 | -50 | -76 | 4 | 17.36 | .000 |
| L Fusiform gyrus |  | -40 | -70 | -12 | 10.93 | .000 |
| L Middle temporal gyrus |  | -50 | -60 | 8 | 10.54 | .000 |
| L Superior frontal gyrus | 2049 | -10 | 50 | 36 | 5.59 | .034 |
| R Posterior cingulate gyrus | 1484 | 2 | -52 | 30 | 6.66 | .002 |
| Precuneus |  | 0 | -50 | 48 | 5.61 | .032 |
| R Precuneus |  | 8 | -52 | 14 | 5.61 | .033 |
| L Orbitofrontal cortex | 1282 | -40 | 24 | -20 | 7.68 | .000 |
| L Ventrolateral prefrontal cortex |  | -52 | 32 | 4 | 5.78 | .021 |
| R Ventrolateral prefrontal cortex | 911 | 54 | 32 | 8 | 9.25 | .000 |
| L Thalamus | 400 | 16 | -28 | 0 | 5.63 | .031 |
| **Low arousal > High Arousal** |  |  |  |  |  |  |
| L Fusiform gyrus | 270 | -34 | -52 | 0 | 6.57 | .003 |
